# Optimal sequencing strategies for identifying disease-associated singletons

**DOI:** 10.1371/journal.pgen.1006811

**Published:** 2017-06-22

**Authors:** Sara Rashkin, Goo Jun, Sai Chen, Genetics and Epidemiology of Colorectal Cancer Consortium (GECCO), Goncalo R. Abecasis

**Affiliations:** 1 Center for Statistical Genetics, Department of Biostatistics, University of Michigan, Ann Arbor, Michigan, United States of America; 2 Department of Epidemiology and Biostatistics, University of California, San Francisco, San Francisco, California, United States of America; 3 Human Genetics Center, School of Public Health, University of Texas Health Science Center at Houston, Houston, Texas, United States of America; 4 Department of Computational Medicine and Bioinformatics, University of Michigan, Ann Arbor, Michigan, United States of America; Montreal Heart Institute, CANADA

## Abstract

With the increasing focus of genetic association on the identification of trait-associated rare variants through sequencing, it is important to identify the most cost-effective sequencing strategies for these studies. Deep sequencing will accurately detect and genotype the most rare variants per individual, but may limit sample size. Low pass sequencing will miss some variants in each individual but has been shown to provide a cost-effective alternative for studies of common variants. Here, we investigate the impact of sequencing depth on studies of rare variants, focusing on singletons—the variants that are sampled in a single individual and are hardest to detect at low sequencing depths. We first estimate the sensitivity to detect singleton variants in both simulated data and in down-sampled deep genome and exome sequence data. We then explore the power of association studies comparing burden of singleton variants in cases and controls under a variety of conditions. We show that the power to detect singletons increases with coverage, typically plateauing for coverage > ~25x. Next, we show that, when total sequencing capacity is fixed, the power of association studies focused on singletons is typically maximized for coverage of 15-20x, independent of relative risk, disease prevalence, singleton burden, and case-control ratio. Our results suggest sequencing depth of 15-20x as an appropriate compromise of singleton detection power and sample size for studies of rare variants in complex disease.

## Author summary

Genetic studies of rare variants can help us understand the biology of human disease. With modern techniques and sufficient effort, it is possible to very accurately resolve any human genome, identifying most of its unique features. When funding is limited, applying these techniques to study human disease often involves a trade-off between examining more samples, at reduced accuracy per sample, or fewer samples, each at greater accuracy. We evaluate these trade-offs for studies of very rare variants, using both simulation and real data. We propose cost effective strategies for increasing our understanding of human disease.

## Introduction

New sequencing technologies are shifting the focus of genetic association studies to rare variants. Rare variants may explain much of the heritability of common, complex diseases [1, 2]. Importantly, trait-associated rare variants are more likely to severely disrupt gene function [1, 3], and can thus accelerate progress from genetic association signals to mechanistic understanding of disease.

It is frequently asserted that the study of rare variants requires deep sequencing, which provides the highest power for variant discovery in any single genome [2, 4]. The alternative of low-pass sequencing has been advocated for studies of common variation [5, 6], supported by empirical studies [7]. Low pass sequencing allows for larger sample sizes but misses some variants in each individual and reduces genotyping accuracy [4, 5]. The 1000 Genomes Project used low pass sequencing of ~2,500 individuals to produce a near complete catalog of common genetic variation and haplotypes across 26 populations, also identifying many rare variants and singletons in the process [6].

We speculated that low pass or intermediate depth sequencing could be useful even for studies of very rare variants. It is now clear that these studies often require large sample sizes, totaling thousands of individuals. Examples of successful rare variant association studies include several studies implicating rare variants in the complement genes (*CFH*, *C3*, *CFI*, *C9*) in the risk of age-related macular degeneration [8–11], a study showing rare *IFIH1* variants protect against type 1 diabetes [12], and a study that found that rare variants that inactivate *NPC1L1* reduce the risk of coronary heart disease [13].

Here, we attempt a more nuanced view of the optimal strategies for sequence based rare variant association studies and explore and compare the power of rare variant association studies that use low, intermediate, or deep sequencing strategies. Since common variants can be detected and genotyped efficiently by analyzing sequence data for many individuals jointly, we focused our analysis on singletons. Any sequencing depth that works well for singletons should provide an upper bound of needed sequencing depth for more common variants, for which low pass data can be analyzed more effectively [5, 14]. In this paper, we examine discrete traits and seek to maximize association study power for a fixed sequencing effort. We consider the balance between power to identify very rare variants (which increases with sequencing depth) and power to identify disease association (which increases with sample size). We explore both simulated data and actual sequence data. We estimate the power of association tests for study designs employing deep sequencing, low pass sequencing, and intermediate strategies across a range of sample sizes, singleton frequencies, disease relative risks, and disease prevalence. Our results show that, for fixed cost, power to detect association is maximized at a read depth of 15-20x and decreases rapidly as coverage is increased beyond this threshold.

## Results

### Sensitivity to detect singletons

**Fig 1:**
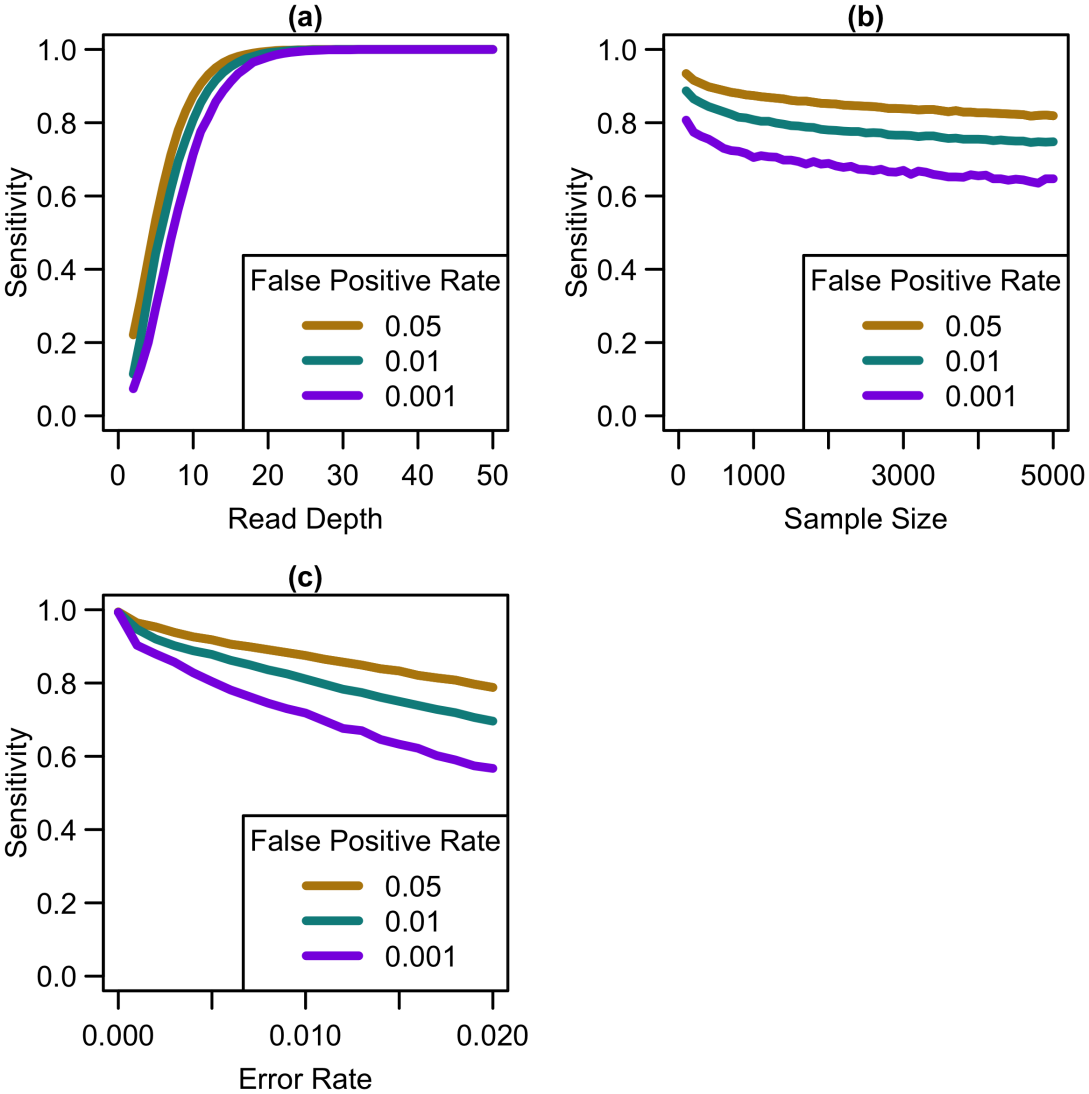
Sensitivity to detect singletons by read depth, sample size, and sequencing error rate. Sensitivity vs. (a) read depth for N = 1000 and e = 0.01, (b) sample size for d = 10x and e = 0.01, and (c) sequencing error rate for N = 1000 and d = 10x at different false positive rates.

Our simulations show that, for a fixed sample size, sensitivity to detect singletons increases rapidly as coverage increases until ~25x (see Fig 1a). After this point, increasing coverage has little effect on sensitivity. As sample size increases for a fixed depth, sensitivity decreases only slightly (see Fig 1b), implying that coverage at a site has more impact than sample size in the overall ability to detect singletons. For constant depth and sample size, an increase in sequencing error rate reduces sensitivity (see Fig 1c). At higher false positive rates, sensitivity is greater (see Fig 1), although the number of incorrectly called singletons increases as well. Among the settings we considered, by 25x, sensitivity reaches 98.6% for a sequencing error rate of 0.005, 96.2% for an error rate of 0.01, and 89.9% for a sequencing error rate of 0.02, regardless of sample size or false positive rate. Increasing depth to 30x resulted in sensitivity of 99.6% for a sequencing error rate of 0.005, 98.8% for an error rate of 0.01, and 95.9% for an error rate of 0.02. Further increasing depth to 50x, resulted in 100% sensitivity, regardless of error rate.

**Fig 2:**
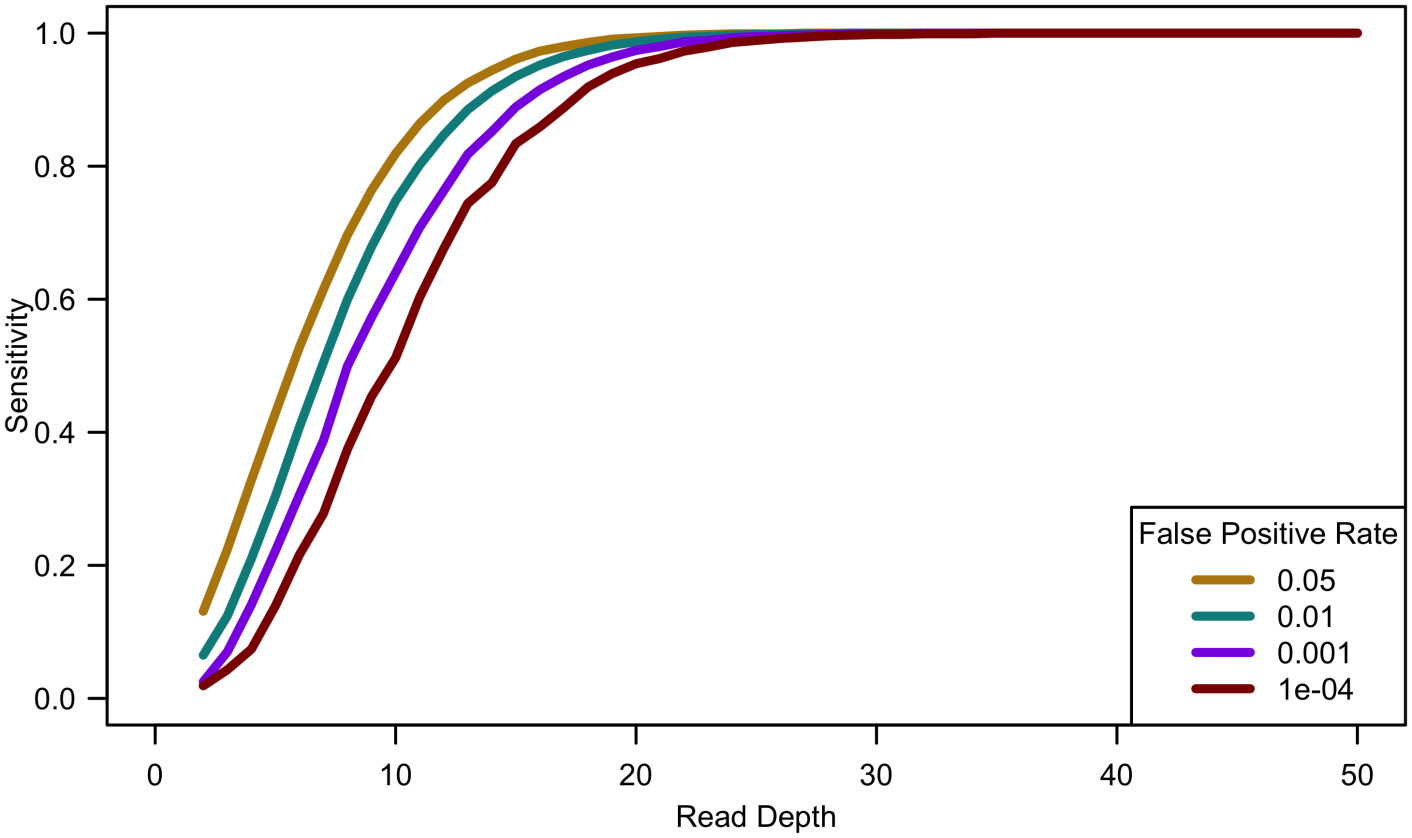
Sensitivity to detect singletons by read depth for constant cost. Comparing computational simulations (for a sequencing capacity of 50,000x) for sensitivity to detect singletons for different false positive rates shows that power increases until 25-30x, exact threshold increasing with increased error rate or decreased false positive rate. (Sample size = cost/depth, assuming no cost of library/sample preparation, *c* = 0).

As shown in Fig 1, variant detection sensitivity changes rapidly with read depth but only very slowly with sample size (sensitivity decreases slightly with increased sample size because, when depth and total false positive rate are fixed, the caller must become gradually more stringent as more samples are sequenced so as to maintain a fixed false-positive rate). We next explored variant discovery power in experiments with constant cost, where sample size and read depth vary in opposite directions. We first considered a simplified case with no additional cost for library and sample preparation, so that read depth and sample size are inversely proportional. In this case, as coverage increased, sensitivity increased until 20-25x, after which increasing read depth had little effect on sensitivity (see Fig 2). When we varied the total sequencing capacity, there was little difference between the sensitivity to detect singletons at a fixed read depth, emphasizing that read depth is more influential than sample size. For instance, at 10x coverage, sequencing 5,000 samples provides 64% sensitivity, sequencing 10,000 samples provides 60.8% sensitivity, and sequencing 20,000 samples provides 57.9% sensitivity; whereas, at 20x coverage, sequencing 5,000 samples provides 97.4% sensitivity, sequencing 10,000 samples provides 96.8% sensitivity, and sequencing 20,000 samples provides 96.1% sensitivity.

**Fig 3:**
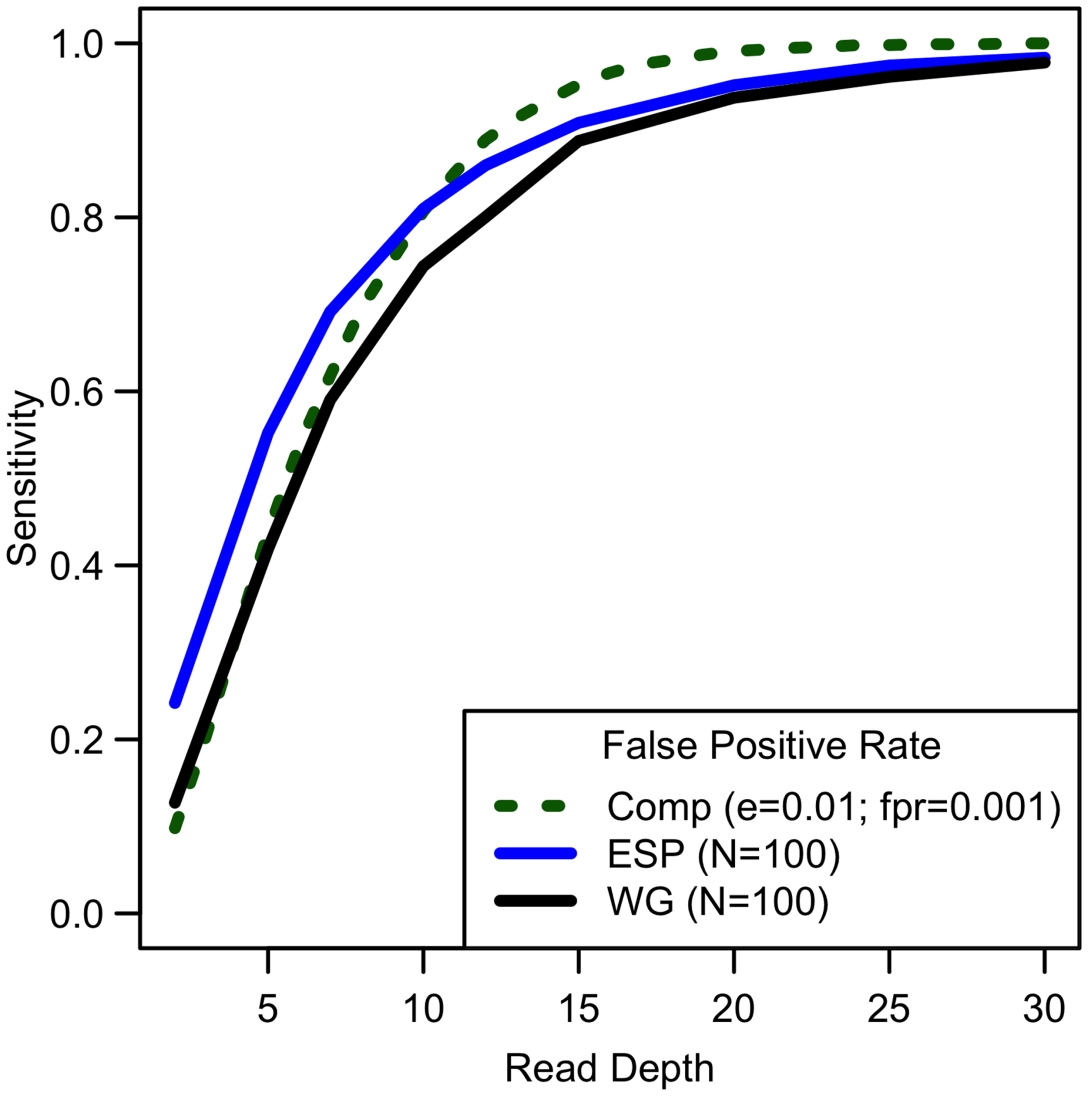
Comparison of empirical sensitivity to detect singletons with computational estimates. For a sample size of 100, sequencing error rate of 0.01 with a false positive rate of 0.001, empirical and computational estimates are similar.

Analyses of down-sampled data validated our computational simulations (see Fig 3). For a fixed sample size of 100 individuals, empirical estimates of sensitivity closely resemble simulations that assume a sequencing error rate of 0.01 and a false positive rate of 0.001, though the simulations were slightly pessimistic at lower depths—detecting a lower proportion of variants than in the down-sampled data—and slightly optimistic at higher depths—detecting a larger fraction of variants than in the down-sampled data (see Fig 3). Potential explanations for these differences include that (a) sequence coverage is less evenly distributed in real data and (b) real data includes a mixture of high and low quality bases, rather than a fixed per base error rate. In experiments that follow, we use *e* = 0.01 and *γ* = 0.001 to estimate variant detection sensitivity and assess association study power for a broad range of cost models and sequencing capacities.

### Power to detect association

**Fig 4:**
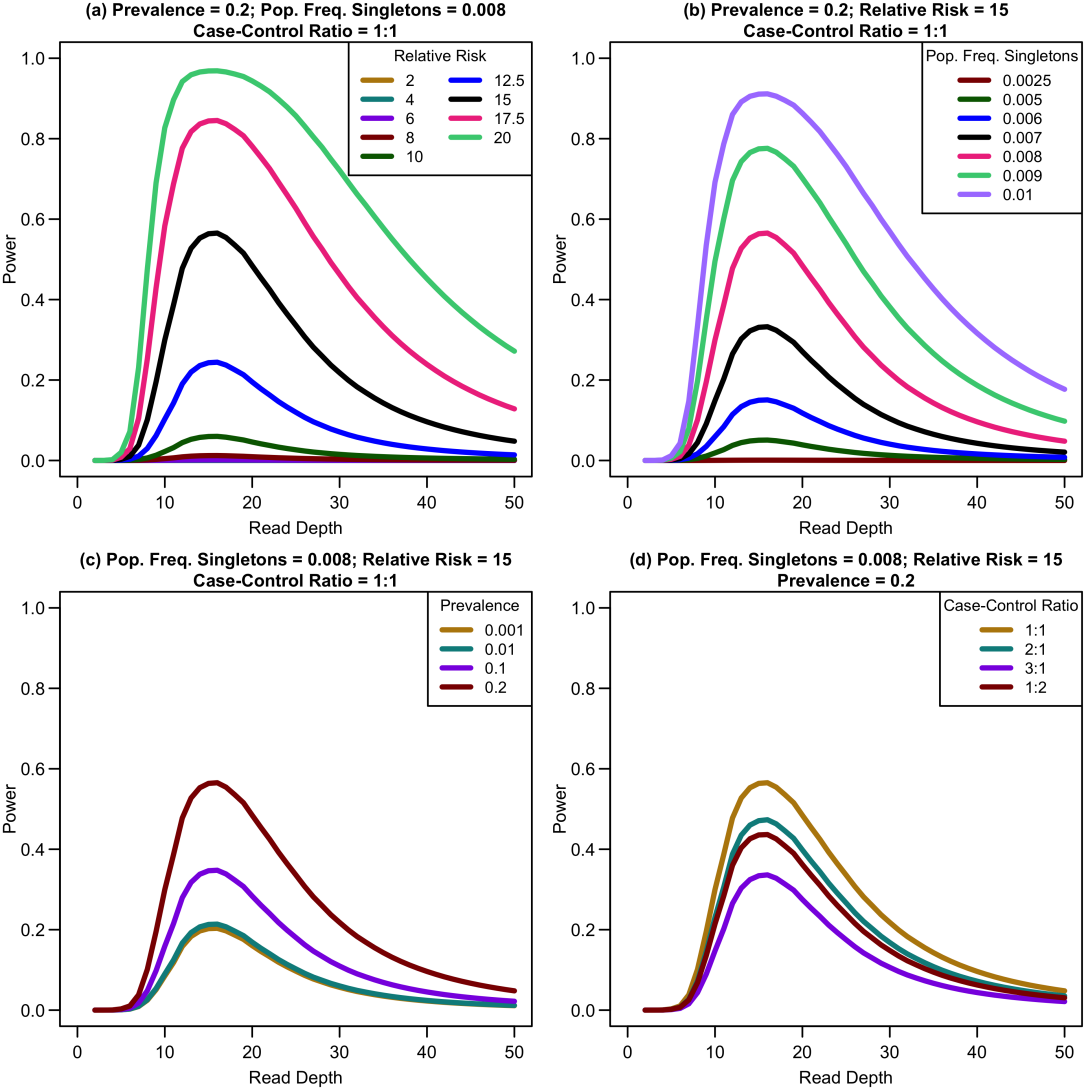
Association study power by read depth for constant cost. Power of an association study increases with relative risk (a), population frequency of singletons (b), prevalence (c), and ratio of cases to controls (d) for a fixed sequencing capacity of 100,000x with no extra cost for library/sample preparation (*c* = 0).

We first considered a situation of fixed cost (sample size and read depth vary inversely) with no extra cost of library/sample preparation (*c* = 0) for equal numbers of affected and unaffected individuals. As depth increases, association study power quickly reaches a maximum and then rapidly decreases (see Fig 4). For example, sequencing 20,000 samples at 5x provides only 1.19% power, sequencing 6,666 samples at 15x provides 91.08% power, and sequencing 2,000 samples at 50x provides 17.71% power for a relative risk of 15, population frequency of singletons 0.01 per person per gene, and a prevalence of 20%. Maximum power increases with relative risk, population frequency of singletons, or prevalence (see Fig 4a–4c). For unequal numbers of cases and controls, power decreases as the case-control ratio moves further away from 1:1 (see Fig 4d).

**Fig 5:**
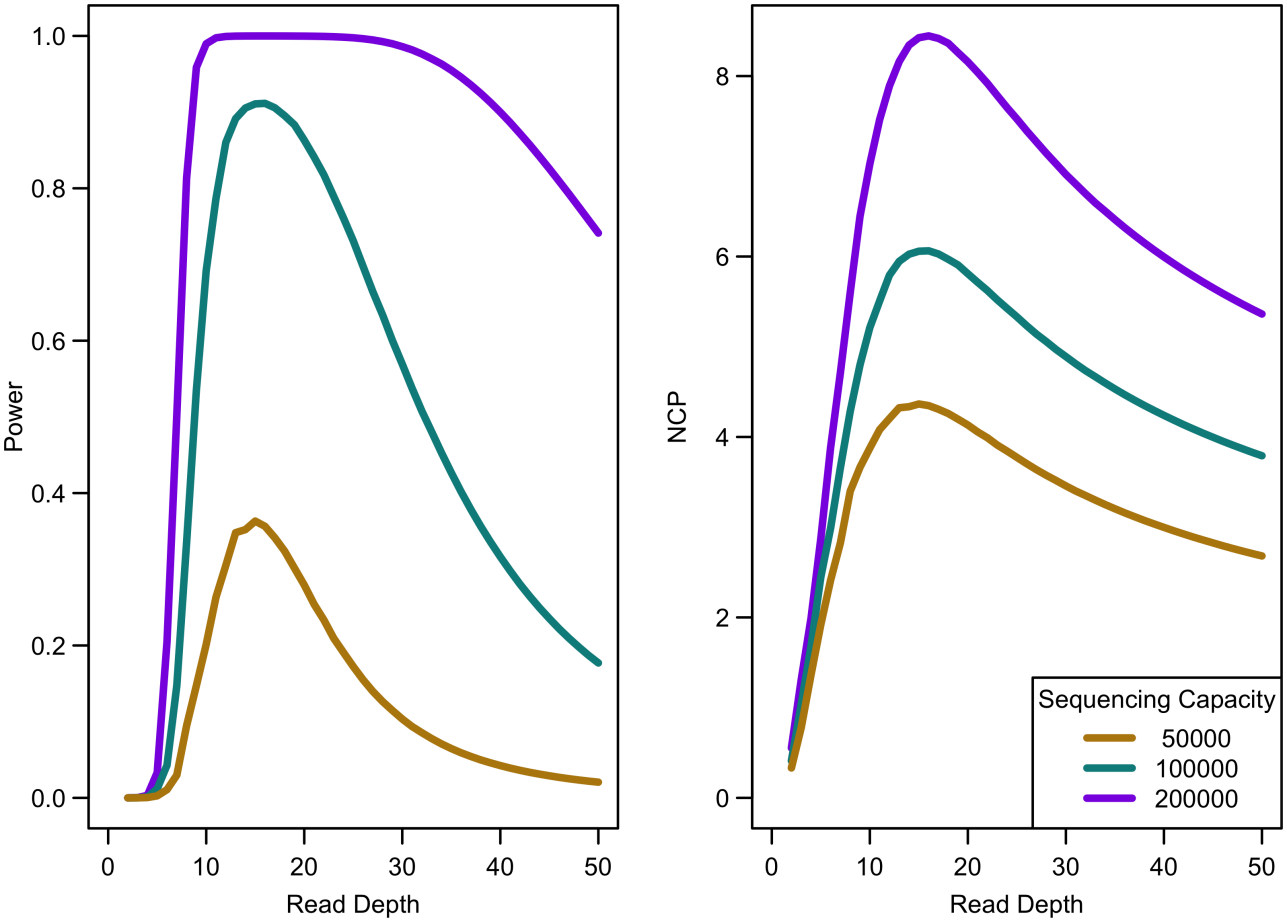
Association study power and NCP by read depth for constant different sequencing capacities. Library/sample preparation costs low (*c* = 0), relative risk 15, population frequency of singletons 0.01, prevalence 20%, case-control ratio 1:1.

**Fig 6:**
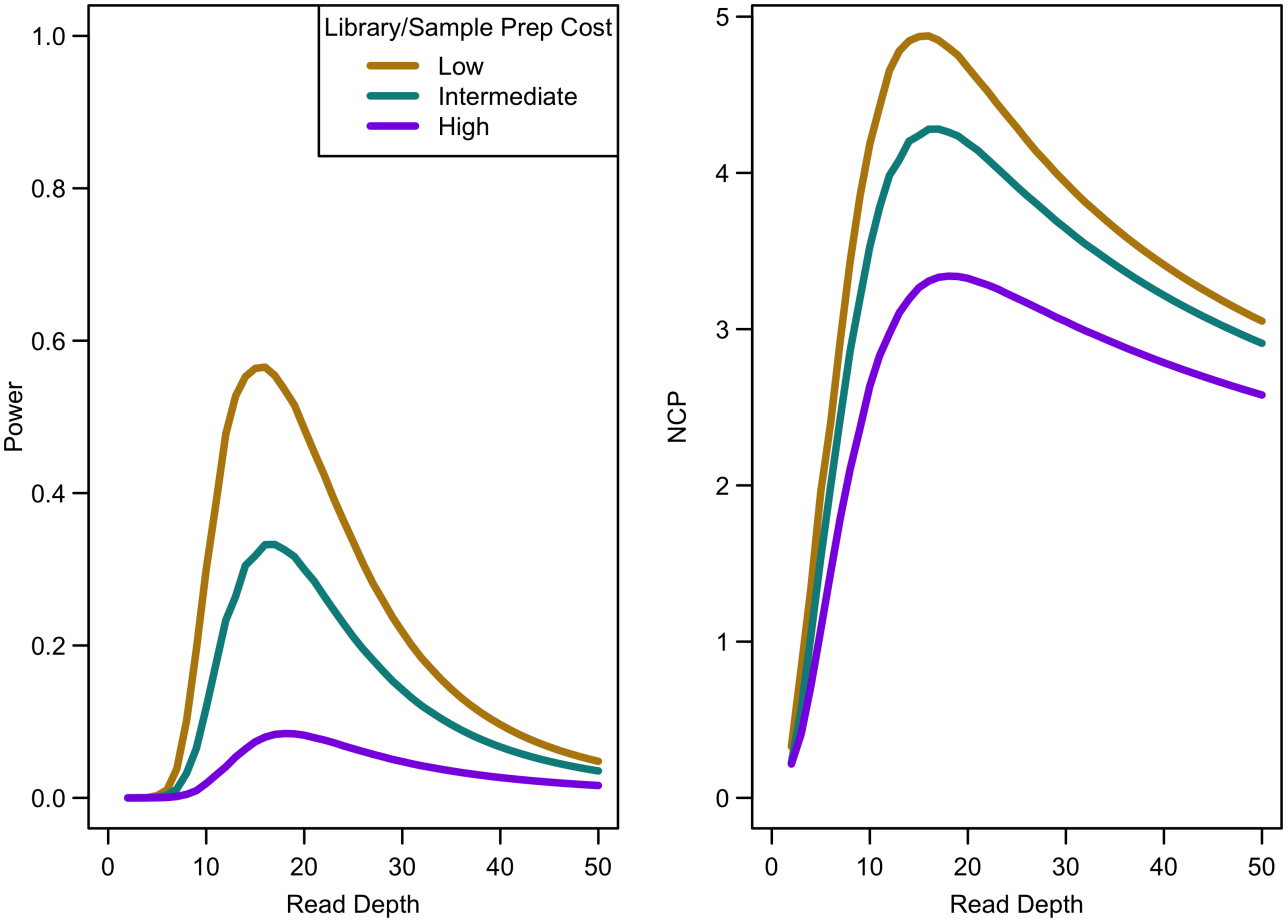
Association study power and NCP by read depth for different sample preparation costs. Sequencing capacity 100,000x, relative risk 15, population frequency of singletons 0.008, prevalence 20%, case-control ratio 1:1.

When the non-centrality parameter (NCP) is large, a change in the NCP might not be reflected in power if the power is already 1. Therefore, we examined the read depth/sample size pair where the maximum NCP, rather than power, was attained. The depth at which the NCP is maximized occurs between 15-20x depending on study cost and the relative cost of library/sample preparation. As available sequencing capacity and total study cost increase, the maximum NCP increases (see Fig 5). As relative cost of library/sample preparation (*c*) increases, NCP decreases slightly (see Fig 6). When either total study cost or *c* increases, the point at which NCP is maximized shifts to a higher depth. For *c* = 0, NCP is maximized at 15-16x; for *c* = 5, NCP is maximized at 16-18x; and for *c* = 20, NCP is maximized at 18-19x. For sequencing capacity = 50,000x, NCP is maximized at 15-18x; for sequencing capacity = 100,000x, NCP is maximized at 16-18x; and, for sequencing capacity = 200,000x, NCP is maximized at 16-19x. This point is not affected much by relative risk, prevalence, population frequency of singletons, or gene length. The overall pattern is easy to understand intuitively: with increasing per sample preparation costs, it is advantageous to sequence fewer samples at higher depth; with increasing total sequencing capacity, the overall sample size increases and a slight increase in sequencing depth is needed to accommodate the greater stringency needed to maintain low false positive rates in variant calling.

**Fig 7:**
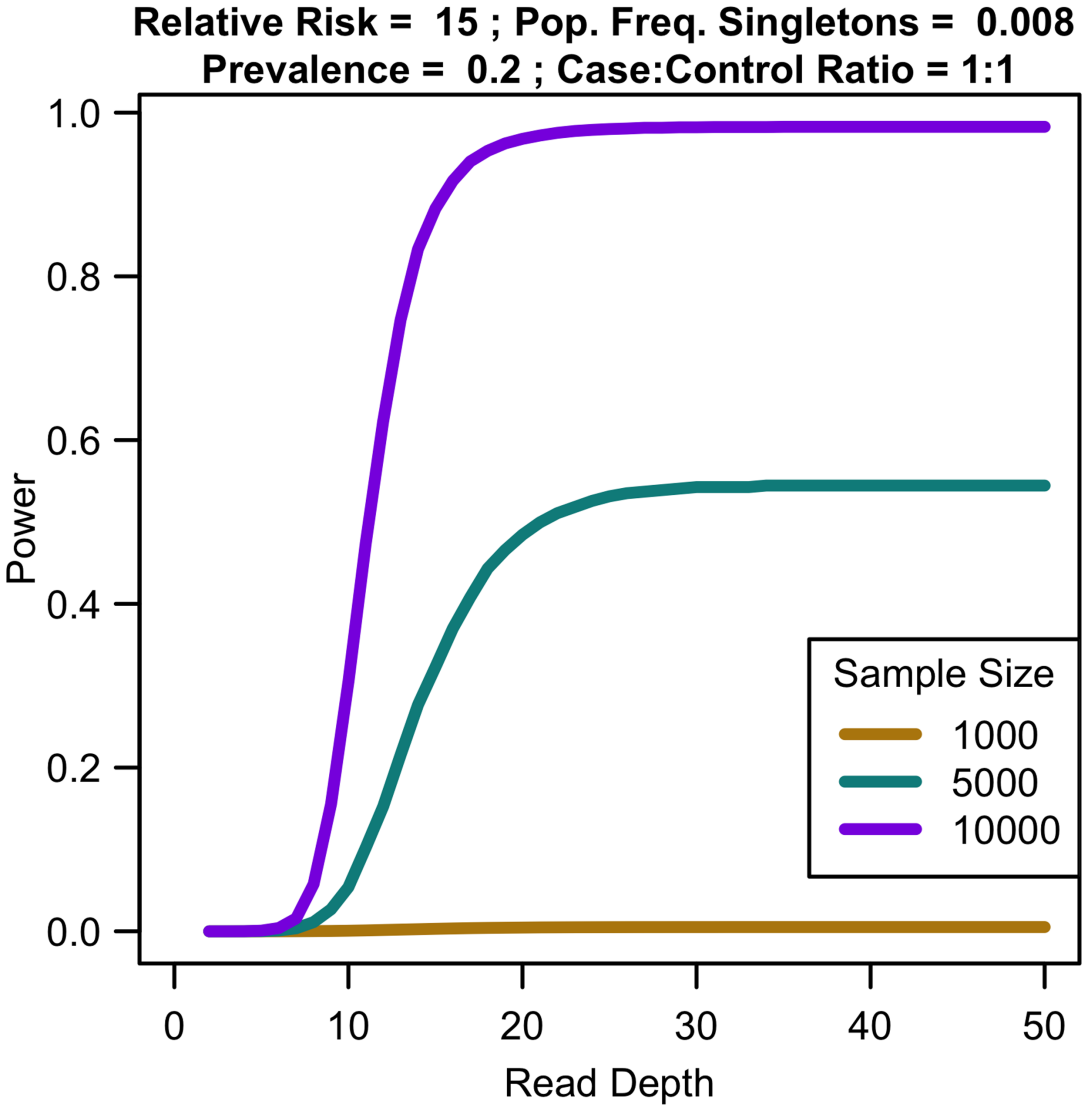
Association study power by read depth for fixed sample size. Increasing coverage beyond a threshold does not increase power of an association study for constant sample size.

For a fixed sample size, increasing coverage is never harmful. At lower depth, increasing coverage increases association study power; at high depths, power eventually plateaus (see Fig 7). For instance, for relative risk of 15, population frequency of singletons 0.008, and prevalence 0.2, sequencing 10,000 samples provides 30.45% power at 10x, 97.99% power at 25x, 98.27% power at 35x, and 98.27% power at 50x. For increased sample size, relative risk, population frequency of singletons, or prevalence, the magnitude of power increases. Regardless of the parameter values, NCP is maximized by 35x, with 99% of maximal NCP occurring by 25x.

### Extending to non-singleton variants

**Fig 8:**
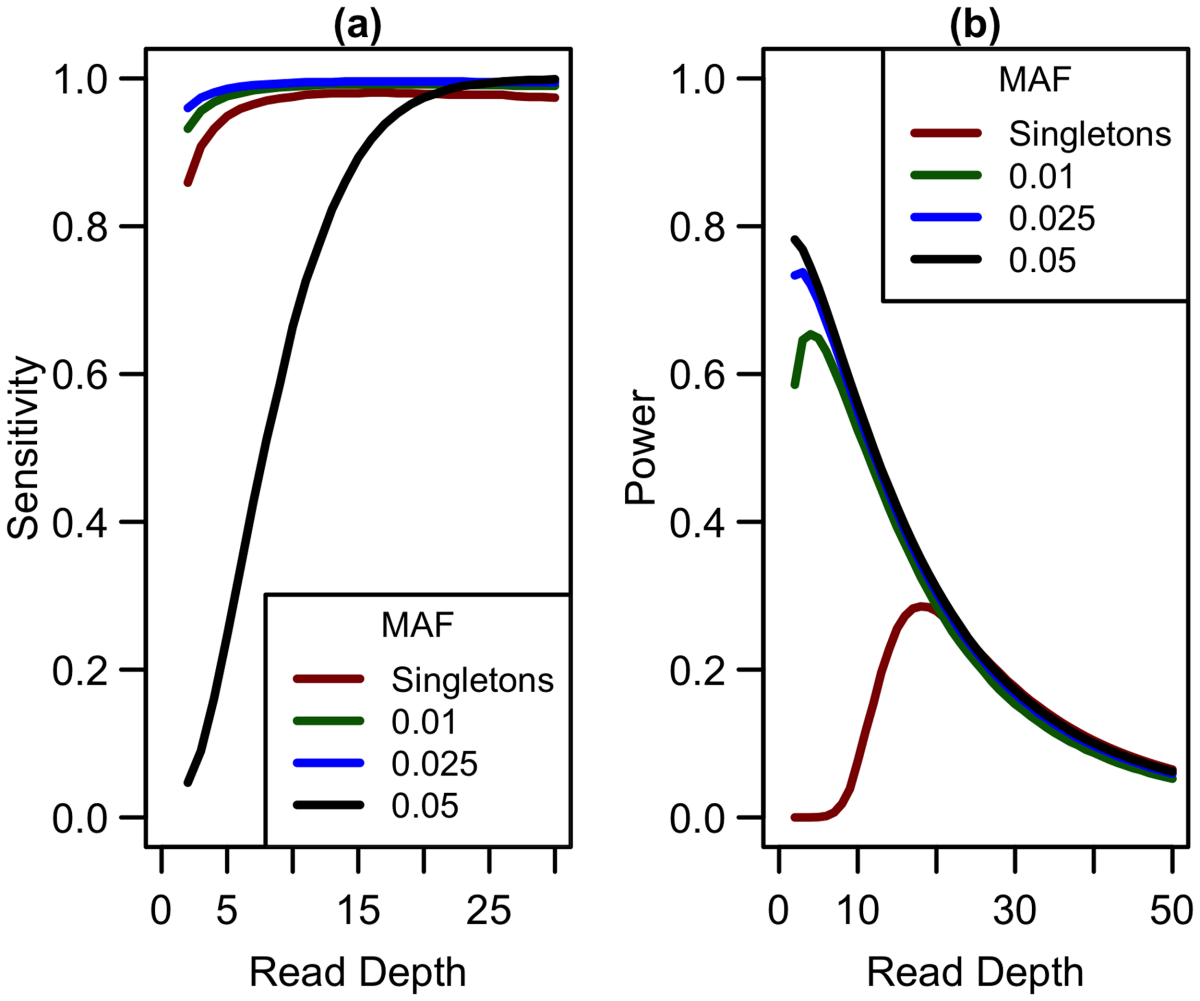
Sensitivity to detect variants and association study power by read depth at different MAF for a fixed sequencing capacity of 100,000x. (a) Sequencing error rate of 0.01 with a false positive rate of 0.001; (b) library/sample preparation costs high (*c* = 20), relative risk 15, population frequency of singletons 0.01, prevalence 20%, case-control ratio 1:1.

Expanding our analysis of rare variants to those more common than singletons, we considered variants with minor allele frequencies (MAF) at thresholds of 0.01, 0.025, and 0.05. Sensitivity to detect variants is lower for singletons than for variants appearing at higher frequencies in the sample (see Fig 8a) when depth is low. As depth increases, sensitivity to detect variants approaches 1 for variants of all frequencies. In terms of detecting association, when considering non-singleton variants, power is maximized at a much lower depth (see Fig 8b) and decreases as read depth increases (and sample size decreases). As sensitivity to detect variants is greater at lower depths, more variants are detected at depths less than 10x, making it preferable to prioritize sample size over depth of coverage. Therefore, if singleton variants are not of interest, sequencing at lower depths (5-10x) may be more optimal.

### Extending to indels

**Fig 9:**
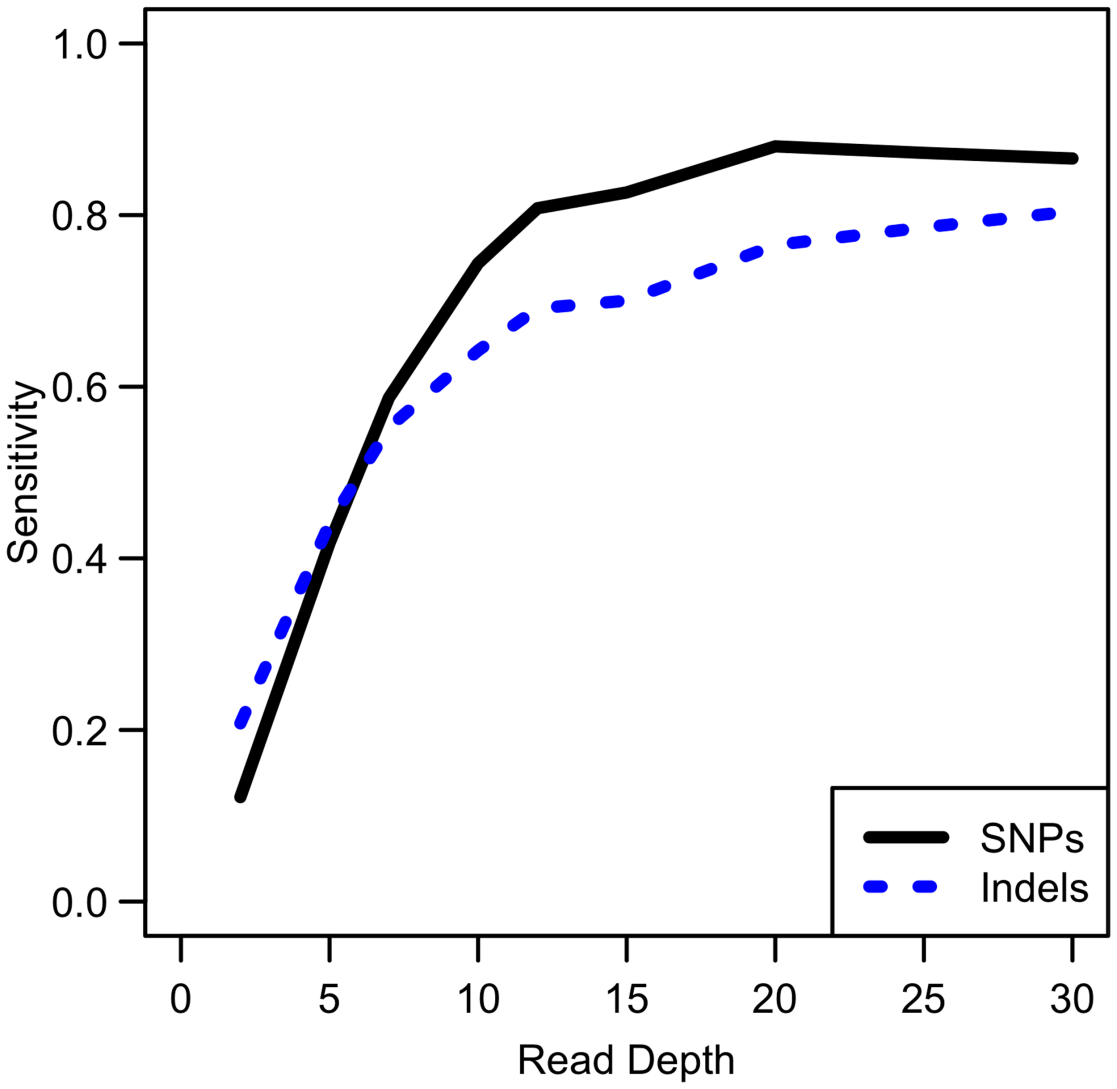
Comparison of empirical sensitivity to detect singleton SNPs and singleton indels. For a sample size of 100 whole genome samples.

While our analysis focuses on SNPs, other types of variants such as insertions and deletions are also of interest. We conducted simulations comparing sensitivity for detecting singleton indels versus singleton SNPs. While our results show that indel variants are more difficult to detect than SNPs (see Fig 9), the sensitivity curve for detecting indels resembles that of SNP detection in that sensitivity reaches a plateau at ~20x. Therefore, association study power likely follows a similar pattern for indels as for SNPs, though power is likely to be reduced for indels due to this reduced sensitivity.

## Discussion

We set out to identify ideal sequencing strategies, in terms of read depth and sample size, focusing on studies exploring the association of singleton variants and discrete traits. We found that association study power is never large unless frequency of singletons or relative risk is large. When cost is fixed so sample size varies inversely of read depth, power decreased as coverage increased beyond 15-20x. Even for fixed sample size, increasing coverage beyond 25x had only a small impact on power. Therefore, we believe it will often be better to sequence larger samples at lower coverage rather than smaller samples at increased coverage when searching for disease associated singletons. We recommend that coverage should only be increased beyond 20x if sample numbers are limited or if applications other than genetic association studies (such as genetic counseling and diagnosis) can justify the advantages of more complete sequencing of each individual at the cost of reduced sample sizes.

While varying prevalence, singleton frequency, and relative risk varies association study power, the combination of read depth and sample size that maximizes power (for a fixed cost) remained constant. For example, consider two scenarios. The first has a relative risk of 10, a prevalence of 20%, and a population frequency of singletons of 0.8%. The other has a relative risk of 12.5, a prevalence of 20%, and a population frequency of singletons of 1%. Both scenarios have a sequencing capacity of 200,000x with intermediate library and sample preparation costs (*c* = 5) and a gene length of 1000 bp. The first scenario attains a maximum power of 19.87% (NCP = 3.86); the second reaches a maximum power of 92.07% (NCP = 6.12). However, in both cases, NCP is maximized at a depth of 18x (with 8,695 samples). In the settings we examined, very deep sequencing is not justified for detecting rare variant association, irrespective of the underlying disease model. While power may be low, increasing coverage beyond a threshold at 15-20x will not increase power if it requires a decrease in sample size. For more common variants, our results show that there is higher sensitivity for detecting variants at lower depths, and association study power will be maximized at even lower coverage.

Of the parameters we considered, only the cost of library and sample preparation changed the depth required to maximize association study power, though this optimal depth remained between 15-20x. For larger library and sample preparation costs, the optimal depth increases slightly. For no extra cost of library/sample preparation (*c* = 0), the ideal depth is 15-16x; for a moderate cost of library/sample preparation (*c* = 5), the ideal depth is 16-18x; and when the cost of library/sample preparation is high (*c* = 20), the ideal depth is 18-19x. For very large studies, the ideal depth shifts to slightly larger depths. For example, when the total sequencing capacity is 50,000x, the ideal depth is 15-18x per genome; when this increases to 100,000x, the ideal depth is 16-18x per genome; and, for sequencing capacity of 200,000x, the ideal depth is 16-19x per genome. This increase in per genome depth allows variant calling to become more stringent as sample size increases (there are more opportunities for false positive calls as more genomes are sequenced).

In summary, we have shown that, while deep sequencing is appealing for detecting a complete catalog of variants, sequencing each sample at lower depth so as to enable increases in sample size results in higher power for association studies, even when these studies focus on rare singletons. When we expand our focus to include other rare variants beyond singletons, higher power for association studies is attained at even lower depth. Additionally, our primary focus was SNPs, but other types of variants such as insertions and deletions are of interest. These non-SNP variants are more difficult to detect than SNPs and may result in lower association study power. An avenue of future work is to conduct a similar analysis for quantitative traits.

## Methods

### Overview

To determine the ideal study design for studying disease-related singletons, we first used simulations to explore the ability to detect singleton variants present in sequenced samples. Next, we validated simulations by comparing results to down-sampled sequence data. Finally, we extended these analyses of variant discovery power to examine association study power.

### Definitions

We consider sequencing studies that assess *N* individuals, sequencing each to an average depth *d*. We assume that the cost of the study is proportional to the summed costs of preparing samples for sequencing, *Nc*, and to the total sequencing depth, *Nd*. (Here, *c* is a constant that places sequencing depth and per sample cost in the same scale).

Thus, we estimate the total cost of a sequencing study as:

$$\begin{array}{ll} Cost & =N\cdot \left(cost\;per\;sample\right)\\ & =N\cdot d\cdot \left(cost\;per\;depth\right)+N\cdot \left(cost\;of\;library\;and\;sample\;preparation\right)\\ & =N\cdot \left(cost\;per\;depth\right)\cdot \left(c+d\right)\\ & \propto N\cdot \left(c+d\right) \end{array}$$

where *N(c+d)* is the total sequencing capacity. In this simple model, to keep total cost constant, sample size and depth must vary inversely of each other (*i*.*e*., if sample size increases, coverage decreases). In our simulations, we first considered *c* = 0. We then expanded our analyses to also consider *c* = 5 and *c* = 20 for total budgets of *sequencing capacity* = 50,000x, 100,000x, and 200,000x. With current genome sequencing costs of $1,000–$3,000 per 30x genome, *c* = 5 and *c* = 20 correspond to costs of ~$250 to $600 and of ~$700–$2,000 for sample collection and preparation, respectively.

### Sensitivity to detect singleton variants

We first used simulations to estimate the sensitivity of singleton discovery. We ran these simulations using different read depths (*d*, ranging from 2x to 50x), sample sizes (*N*, ranging from 100 to 5,000), sequencing error rates (*e*, ranging from 0.001 to 0.02) and singleton discovery false positive rates (*γ*, 0.00001, 0.0001, 0.001, 0.01, and 0.05). For each combination of parameters, we generated 200,000 replicate samples, each with a single individual carrying the simulated singleton variant (when estimating sensitivity) or no individuals carrying the variant (when estimating false positive rates).

We assumed read depth followed a Poisson distribution. For each individual, we track the total number of sequencing reads as well as the number of reads in which a variant base was observed. When simulating data, per individual sequence depth was generated from *Poisson(d)*, and each read was generated assuming sequencing error probability *e*. Briefly, our simulation proceeded as follows. We first sampled a total read depth for each individual and set the number of variant reads to zero. Then, for each read, we select a template chromosome at random. Then, we increased the number of variant reads whenever the simulated read was assigned to a chromosome that has the singleton variant and there is no a sequencing error (probability 1—*e*) or when the simulated read was assigned a chromosome without the variant but there is a sequencing error (probability *e*). We then estimate the likelihood of the observed count of variant reads, conditional on depth, sequencing error rates and an allele frequency; first, assuming that all individuals match the reference so that the number of variant reads in each individual is distributed as Binomial(Prob = *e*, Count = *depth*); next assuming that a rare variant is segregating with frequency *1/2N*, so that the number of variant reads is distributed as (1–*1/2N*) x Binomial(Prob = *e*, Count = *depth*) + *1/2N* x Binomial(Prob = 0.5, Count = *depth*). Finally, we take the ratio of these two likelihoods. The sensitivity for detecting a variant with false positive rate *γ* was computed as the fraction of simulations with a simulated singleton for which the likelihood ratio was greater than the (1- *γ*)^th^ percentile of null simulations using the same sample size, depth, and sequencing error rate parameters.

We validated these estimates by down-sampling on chromosome 20 from deep genome and exome samples and assessing the sensitivity to detect singletons called when all available sequence data was analyzed. Exome samples are from the NHLBI Exome Sequencing Project [1, 15] (original depth of exome sequenced regions in chromosome 20 averaging 106.80x, range 27.10–515.25x). We excluded samples with an average depth <50x to allow for down-sampling to depths 2-50x. Whole genome samples are from the Genetics and Epidemiology of Colorectal Cancer Consortium [16] (original depth averaging 35.79x, range 30.35–42.59x). For whole genome samples, we considered down-sampled depths 2-30x. In each down-sampling analysis, we sampled reads from each individual to create a new sample with the desired average depth. For instance, for an individual with an original depth of 100x, we would retain each of the original reads with a probability of 10% to achieve depth 10x.

After down-sampling, we performed variant calling using SAMtools mpileup [17]. Sensitivity for each subsample was computed as the proportion of singletons in the original deep sequence data that were called in the down-sampled data. The false positive rate was estimated as the proportion of sites where variants were called in the down-sampled data but not in the original deep sequence data. We averaged the results of 100 replications for 100 individuals at each depth. Each exome replicate examined 788,942 bases on chromosome 20 and included an average of 1.70 singletons per person (SD = 0.15); each whole genome replicate examined 63,025,520 bases on chromosome 20 and included an average of 725.09 singletons per person (SD = 7.52).

We chose parameter settings for computational simulations so that results closely mimicked those for analysis of down-sampled real data. We then used these values in the analysis of association study power across a broad range of sample sizes, sequencing depths, and cost models.

### Power to detect association

We estimated association study power analytically by comparing the burden of singletons in a region between cases and controls, at significance level *α* = 2.5x10^-6^, corresponding to the analysis of ~20,000 independent gene regions. Power for a two-sample t-test can be estimated using a non-central t-distribution to model test statistics as a function of sample size, the frequency of singleton variants per gene per person, the increased risk of disease conveyed by a singleton variant, and the sensitivity to detect each singleton (which is a function of sequencing depth).

Modeling this non-central t-distribution requires estimates of a non-centrality parameter λ, which describes the expected value of the statistic for a given disease model and experimental design. The requisite non-centrality parameter *λ* for a two-sample t-test can be expressed as:

$$\lambda =\frac{\mu_{A}-\mu_{U}}{\sqrt{\sigma_{A}^{2}/N_{A}+\sigma_{U}^{2}/N_{U}}}$$

where *μ*_A_ is the mean number of singletons per gene per person in affected individuals, and *σ*_*A*_^*2*^ is the corresponding variance. Similarly, *μ*_*U*_ and *σ*_*U*_^*2*^ are the mean and variance for unaffected individuals. *N*_*A*_ and *N*_*U*_ are the number of affected and unaffected individuals, respectively.

We assume that the number of singletons occurring per gene per person follows a Poisson distribution with rate parameter equal to the product of gene length (*L*) and frequency of singleton occurrence per site per person for cases (*p*_*A*_) or controls (*p*_*U*_). For a subset of simulations, we compared results of a two-sample t-test and a Wilcoxon rank-sum test. Since both gave similar results, we proceeded with the two-sample t-test.

The non-centrality parameter can be expanded as:

$$\lambda =\frac{L\cdot p_{A}-L\cdot p_{U}}{\sqrt{L\cdot p_{A}/N_{A}+L\cdot p_{U}/N_{U}}}$$

Here, *p*_*A*_ and *p*_*U*_ are the cumulative frequencies of singletons among cases and controls (for deeply sequenced samples). For low and intermediate sequencing depths, we replace these with *p*_A_* and *p*_U_*, which are the frequency of detected singletons in cases and controls at a given sequencing depth. These quantities can be defined as:

$$\begin{array}{ll} p_{A}^{*} & =P\left(detect\;singleton|d,\;N,\;case\right)\\ & =P\left(detect\;singleton|singleton,\;d,\;N\right)\cdot P\left(singleton|case\right)\\ & +\;P\left(detect\;singleton|no\;singleton,\;d,\;N\right)\cdot P\left(no\;singleton|case\right) \end{array}$$

$$\begin{array}{ll} p_{U}^{*} & =P\left(detect\;singleton|d,\;N,\;control\right)\\ & =P\left(detect\;singleton|singleton,\;d,\;N\right)\cdot P\left(singleton|control\right)\\ & +\;P\left(detect\;singleton|no\;singleton,\;d,\;N\right)\cdot P\left(no\;singleton|control\right) \end{array}$$

where *P(detect singleton* | *singleton*, *d*, *N)* is the sensitivity to detect singleton variants for a given read depth and sample size, and *P(detect singleton* | *no singleton*, *d*, *N)* is the corresponding false positive rate. The frequency of singleton occurrence in cases and controls can be expressed as:

$$P\left(singleton|case\right)=\frac{prf/L}{prf/L+\left(1-p/L\right)f}=\frac{pr/L}{pr/L+\left(1-p/L\right)}$$

$$P\left(singleon|control\right)=\frac{p/L\left(rp/L+1-p/L-rf\right)}{\left(1-f\right)\left(rp/L+1-p/L\right)}$$

where *r* is relative risk of disease (ranging from 2 to 20 in our simulations), *p* is population frequency of singletons (ranging from 0.001% to 1% per gene per person in our simulations), *L* is gene length (ranging from 1,000 to 50,000 bps in our simulations), and *f* is the background prevalence of disease (ranging from 0.1% to 20% in our simulations). We considered different case-control ratios (1:1, 2:1, 3:1, and 1:2 in our simulations).

We considered prevalences from 0.1% to 20% to explore scenarios for studying different diseases. Such diseases include very complex diseases such as cardiovascular disease, which has a prevalence of 33% in American adults [18], intermediate frequency diseases such as age-related macular degeneration, which has an estimated prevalence of 1.47% in Americans 40 years and older [19], but also less common diseases such as type 1 diabetes, which has an approximate prevalence of 0.33% in Americans 18 years and younger [20].

From our exome samples, we estimated that singletons occur at a rate of 0.79% per gene per person for the entire genome (assuming approximately 20,000 genes). This rate fluctuates when looking at specific genes. Longer genes are more likely to include singletons than shorter genes. For instance, *AVP* (a gene that provides instructions for making the hormone vasopressin) is 2,169 bp long [21] and has an estimated frequency of singletons of 0.058% per person in the coding region, while *TTN* (encoding a giant muscle protein that plays a key role in muscle assembly and one of the longest genes in the human genome) is 281,435 bp long [22] and has a singleton frequency of 42% per person in the coding region. Genes of different functions might have different frequencies of singletons. We varied population frequency of singletons from 0.001% to 1% per gene per person to account for this wide set of possibilities.

Once the non-centrality parameter is computed, the power of an association test can be estimated by:

$$Power=P\left(\left|X\right|>t_{\alpha /2}\left(\nu ,\;0\right)|X\sim t\left(\nu ,\;\lambda \right)\right)$$

where

$$\begin{array}{ll} t_{\alpha /2}\left(\nu ,\;0\right) & =100\left(1-\alpha /2\right)\;percentile\;of\;the\;central\;t\;with\;\nu \;degrees\;of\;freedom\\ t\left(\nu ,\;\lambda \right) & =Non-central\;t\;with\;\nu \;degrees\;of\;freedom\;and\;non-centrality\;parameter\;\lambda \end{array}$$
